# Supplementary material for: Soil copper sources and biogeochemical processes under different land uses: insights from stable copper isotopes in the Cambisols of Southwest China
Source: PeerJ. 2025 Sep 2;13:e19982. doi: 10.7717/peerj.19982 (PMC12422267; doi:10.7717/peerj.19982)
Supplement: Supplemental Information 2 [file peerj-13-19982-s002.docx]

| Sampling location | Longitude and latitude | Mean annual precipitation (mm) | Climate | Soil type | Soil δ^65^Cu value (‰) | Source |
| --- | --- | --- | --- | --- | --- | --- |
| Alsatian foothills, Somalia | 47°57’N, 7°17’E | 600 | Subhumid climate | Cambisol | 0, 0.0604, 0.0518, 0.0216, 0.1101, 0.0518, 0.0820, 0.2396, 0.1295, –0.1187, 0, 0.0296, 0.0296, 0.0394, 0.0310, 0.0197, 0.0803, –0.0507, 0.0197 | Babcsányi et al., 2016 |
| Krompachy, Slovakia | 48°54’N, 20°52’E | 700 | Subhumid climate | Cambisol | 0.19, 0.11, 0.17, –0.12, 0.16, 0.09, –0.05, 0.18, 0.36, 0.19, 0.16, 0.1, 0.01, 0 | Bigalke et al., 2010 |
| Idstein, Germany | 50°13’N, 8°16’E | 1100 | Humid climate | Cambisol | –0.34, –0.23, 0.08, –0.13, –0.06, 0.02, –0.09 | Bigalke, Weyer & Wilcke, 2011 |
| Soave wine region, northern Italy | 45°25’N, 11°14’E | 800 | Humid climate | Cambisol | 0.28, 0.21, 0.25, 0.31, 0.37, 0.31, 0.26, 0.12, 0.22, 0.14, 0.16, 0.16, 0.21, 0.17, 0.19 | Blotevogel et al., 2018 |
| France | 24°N—51°N, 5°W—8°E | 641 | Subhumid climate | Cambisol | –0.15, 0.09, 0.01, 0.13, –0.2, –0.07 | Fekiacova, Cornu & Pichatet, 2015 |
| Huaibei province, China | 33◦36’N—33◦ 40’N, 116◦34’E—116◦44’E | 956 | Humid climate | Cambisol | 0.0690, 0.0989, 0.1218, 0.1494, 0.1747, 0.2276, 0.2414, 0.2713, 0.3011 | Ren et al., 2022 |
| Karst region, southwestern China | 26°15’N, 105°47’E | 1315 | Humid climate | Cambisol | –0.1736, –0.1584, –0.1433, –0.1850, –0.4196, –0.0091, 0.0950, 0.0360, 0.0632, –0.0736, 0.0287, 0.2047, 0.0436, –0.0506, –0.1357, –0.0513, –0.0901, –0.0240, –0.0052, –0.1292, 0.3381, 0.2475, –0.3648, –0.6271, –0.5889 | This study |
